# In-Hospital Macro-, Meso-, and Micro-Drivers and Interventions for Antibiotic Use and Resistance: A Rapid Evidence Synthesis of Data from Canada and Other OECD Countries

**DOI:** 10.1155/2022/5630361

**Published:** 2022-03-16

**Authors:** Rosa Stalteri Mastrangelo, Anisa Hajizadeh, Thomas Piggott, Mark Loeb, Michael Wilson, Luis Enrique Colunga Lozano, Yetiani Roldan, Hussein El-Khechen, Anna Miroshnychenko, Priya Thomas, Holger J. Schünemann, Robby Nieuwlaat

**Affiliations:** ^1^Department of Health Research Methods, Evidence, and Impact, McMaster University, Hamilton, ON, Canada; ^2^Departments of Pathology and Molecular Medicine, McMaster University, Hamilton, ON, Canada; ^3^Michael G. DeGroote Institute for Infectious Disease Research, McMaster University, Hamilton, ON, Canada; ^4^Michael G. DeGroote Cochrane Canada and GRADE Centre, McMaster University, Hamilton, ON, Canada; ^5^Department of Medicine, McMaster University, Hamilton, ON, Canada

## Abstract

Hospitals continue to face challenges in reducing incorrect antibiotic use due to social and cultural factors at the level of the health system, the care facility, the provider, and the patient. The objective of this paper is to highlight the social and cultural drivers of antimicrobial use and resistance and targeted interventions for secondary and tertiary care settings in Canada and other OECD countries. This paper is an extension of the synthesis conducted for the Public Health Agency of Canada's 2019 Spotlight Report: Preserving Antibiotics Now and Into the Future. We conducted a systematic review with a few modifications to meet rapid timelines. We conducted a search in Ovid MEDLINE and McMaster University's evidence databases for systematic reviews and then for individual Canadian studies. To cast a wider net, we searched OECD organization websites and screened reference lists from systematic reviews. We synthesized the evidence narratively and categorized the evidence into macro-, meso-, and microlevel. A total of 70 studies were (a) from OCED countries and summarized evidence of potential sociocultural antimicrobial resistance and use barriers or facilitators and/or interventions addressing these challenges; (b) systematic reviews with 50% of included studies that are situated in secondary and tertiary settings; and (c) published in Canada's two official languages, English and French. We found that hospital structures and policies may influence antibiotic utilization and variations in antimicrobial management. Microlevel factors may sway inappropriate prescribing among clinicians. The amount and type of antibiotics used may affect resistance rates. Interventions were mainly comprised of antibiotic stewardship and training that modify clinician behavior and that educate patients and carers. This evidence synthesis illustrates the various drivers of, and interventions for, antimicrobial use and resistance at the macro-, meso-, and microlevel in secondary and tertiary settings. We demonstrate that upstream drivers may lead to downstream events that influence antimicrobial resistance.

## 1. Introduction

Inappropriate use of antimicrobials and the lack of new classes of antibiotics have reduced our ability to manage antimicrobial or antibiotic resistance (AMR or ABR) [1, 2]. Despite previous efforts to manage AMR through infection prevention and control practices and antimicrobial stewardship programs, AMR is threatening global health and the world's sustainable development goals [3]. A review revealed that, by the year 2050, countries in the Organization for Economic Cooperation and Development (OECD) may experience cumulative losses of 2.9 trillion USD due to AMR [4, 5]. In 2018, AMR reduced Canada's gross domestic product by $2.8 billion. By 2050, this number is projected to increase to $13 billion or more if resistance rates increase [6]. In response to this global health crisis, the United Nations and its member states committed to tackle AMR sustainably and across the human, animal, and environmental reservoirs [7, 8].

Appropriate patient care within hospitals relies on the use of effective antibiotics. When exposed to an antibiotic, bacteria become subject to “selective pressure,” which means that susceptible bacteria will be eradicated but bacteria that are resistant will have a competitive advantage and will be able to replicate and survive [9]. Although a natural phenomenon, inappropriate antibiotic use is a key factor in the development of AMR. In 2015, the death toll due to AMR among countries of the European Union and European economic area reached 33,000 people, and 75% of these deaths were attributed to health care associated infections (or nosocomial infections) [10]. Hospital budgets are also affected by AMR—compared with antibiotic-susceptible infections, resistant infections can cost hospitals an additional 10,000 to 40,000 USD per year [4].

Much of the World Health Organization (WHO)'s critical and important AMR priority areas to support research and development of antibiotics relate to nosocomial bacteria that tend to increase length of stay and mortality within hospitals [11]. Reducing antimicrobial or antibiotic use (AMU or ABU) in hospitals is correlated with reductions in the emergence of resistant nosocomial conditions, including methicillin-resistant *Staphylococcus aureus* and vancomycin-resistant *Enterococcus* [12]. However, hospitals continue to face challenges in reducing inappropriate AMU/ABU due to several social and cultural factors at the level of the health system, care facility, provider, and patient.

The social determinants of health consider the way people live, work, and grow, as well as societal cultures, and how these influence health and well-being [13, 14]. Managing AMR requires an understanding of the various social and cultural factors that drive inappropriate use including health care practices, access to resources, hierarchies among health care workers, and structural issues [15].

Organizing health care systems into various strata (or levels)—“macro, meso, and micro”—may clarify the complexities of the drivers of AMU/ABU and AMR/ABR. The macrolevel refers to national/regional systems and policies, the mesolevel signifies the health care organization and hospitals/hospital wards, and the microlevel represents the individuals including patients, carers, and clinicians. Such a framework may inform and design interventions, programs, and initiatives that target these drivers and appreciate the interactions that exist within and across these levels [16].

Interventions, including antibiotic stewardship programs (ASPs), have been used at the macrolevel and mesolevel to optimize and control AMU and downstream, at the individual level, among health care workers. The role of ASPs is mainly to monitor AMU, develop prescription targets and surveillance, promote infection prevention and control, and modify behaviors, as well as education and awareness of AMR [15, 17]. Drug-resistant infections pose a significant economic burden on health care systems and threaten hospitalized patients and the community [18]. We undertook a commissioned rapid evidence synthesis at the Michael G. DeGroote Cochrane Canada and GRADE Centres to inform the Chief Public Health Officer of Canada's Spotlight Report published June 2019, Handle with Care: Preserving Antibiotics Now and Into the Future [19]. This manuscript is an extension to the 2019 Spotlight Report and is centred on AMU and AMR in secondary and tertiary care settings.

## 2. Objectives

The objectives were to summarize evidence from secondary and tertiary health care settings regarding sociocultural drivers of, and targeted interventions for, antimicrobial use (including antibiotic use), as relevant to Canada and other OECD countries. An accompanying paper draws from the same rapid evidence synthesis; however, it is centred on primary and community care settings. We separated studies from these two settings to elucidate the varying drivers and interventions.

## 3. Methods

### 3.1. Protocol and Registration

Between February and April 2019, we undertook a rapid evidence synthesis at the Michael G. DeGroote Cochrane Canada and GRADE Centres, commissioned by the Public Health Agency of Canada. Our aim was to identify the social and cultural drivers of, and targeted interventions for, antimicrobial use and resistance. Although the Public Health Agency of Canada's report focused on primary care settings, our search included evidence on secondary and tertiary care settings. This paper synthesizes the evidence from secondary and tertiary care settings, which includes hospital wards, emergency departments, and hospital inpatient pharmacies. The protocol for this rapid evidence synthesis is registered in PROSPERO (registration #: CRD42019129669). This paper is reported according to the Preferred Reporting Items for Systematic Review and Meta-Analysis (PRISMA) guidelines.

### 3.2. Eligibility Criteria

We focused on studies from OECD countries that summarized evidence for potential sociocultural AMR/AMU barriers or facilitators and/or interventions addressing these challenges. We included systematic reviews if >50% of included studies were situated in secondary and tertiary settings: hospital wards, emergency departments, and hospital inpatient pharmacies. We placed language restrictions to strictly Canada's two official languages, English and French, given the Canadian focus. Given our rapid timelines, we selected articles that were published in 2009 or later for systematic reviews only. We also included individual Canadian studies to summarize the evidence from the Canadian context given the overall goal to inform Canadian public health regarding AMR and to potentially address these issues in Canada [20]. This is why we did not put restrictions on studies published in the Canadian context. We acknowledge that there are detailed frameworks to assess social, cultural, and other contextual factors; however, we did not pursue the same level of detail as those of these frameworks given our timelines to complete this rapid review.

### 3.3. Search Strategy

We searched for evidence in Ovid MEDLINE (from inception to February 23, 2019) for existing systematic reviews and then for individual Canadian studies (from inception to March 8, 2019) using a hierarchical approach. We also searched McMaster University's evidence databases (ACCESSSS–MD+, Health Evidence, and Health Systems Evidence), OECD organizations websites, and references from systematic reviews for individual Canadian studies (Supplementary Materials).

Reviewers independently and in duplicate screened titles and abstracts and resolved disagreements by consensus. We undertook full-text screening and data extraction concurrently and included studies that could still be excluded at the data extraction or analysis stage if deemed ineligible by two reviewers. We worked closely with the Public Health Agency of Canada and crossed referenced our search results to ensure that we captured relevant studies.

### 3.4. Data Collection

Reviewers independently extracted the data using a pilot tested and revised form, and a senior author confirmed the extraction. We recorded systematic review and study characteristics, results, and main conclusions. For grey literature, we extracted information on policies, guidelines, AMR/AMU goals, research programs, and their evaluations. Reviewers created summary of findings tables to organize evidence per outcome (Supplementary Materials).

### 3.5. Risk of Bias Assessment and Quality Appraisal

We used the risk of bias assessment provided by the systematic reviews, if they were available. We did not assess the risk of bias of the systematic review or the original studies using standardized tools given our rapid timelines. All reviewers were trained in using the Risk of Bias in Systematic Reviews (ROBIS), the Cochrane risk of bias tools for RCTs, the Newcastle-Ottawa Scale (NOS), the Risk of Bias in Non-randomized Studies-of Interventions (ROBINS-I), and the Grading of Recommendations Assessment, Development and Evaluation (GRADE).

### 3.6. Synthesis of the Evidence

Reviewers used a narrative synthesis to analyze the evidence from quantitative, qualitative, and mixed methods studies. We organized studies by outcome, from which two themes emerged: we identified outcomes that relate to antimicrobial use (prescription uptake, guideline adherence) and antimicrobial resistance (prevalence, stewardship). We used a framework (Table 1), developed a priori based on previous knowledge of AMR/ABR and AMU/ABU, to further stratify the evidence into the various levels of our health care systems including macrolevel (national and regional systems and policies), mesolevel (health care organization and hospitals/hospital wards), and microlevel (individuals: patients/carers and clinicians).

Authors independently, and in duplicate, used data extraction forms and developed the summary of findings tables to categorize each study into our two themes: antimicrobial use and antimicrobial resistance. We identified the most appropriate level (macro-, meso-, or microlevel) and outcome(s) reported for each study. Disagreements were resolved through discussion. For studies that fell into multiple levels and outcomes, the results were separated accordingly. Within each level and outcome, we summarized and grouped the common results and the themes wherever appropriate.

## 4. Results

**Figure 1:**
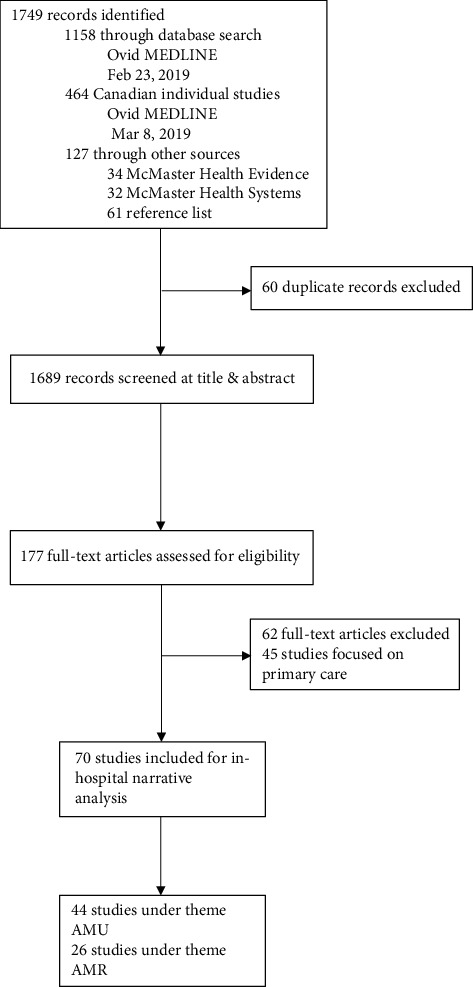
PRISMA flow diagram of study selection. AMU: antimicrobial use; AMR: antimicrobial resistance.

Reviewers screened through a total of 1689 records, of which 177 studies were deemed eligible for full-text screening and data extraction. Of these, 70 studies met inclusion criteria and focused on secondary and tertiary care settings including emergency departments (ED), intensive care units (ICUs), teaching hospitals, and hospital laboratories: 44 studies were categorized under the theme of antimicrobial use and 26 studies were categorized under the theme of antimicrobial resistance (Figure 1). The study designs that are included are systematic reviews, before-and-after studies, cross-sectional studies, randomized control trials, observational studies, retrospective cohorts, prospective audit and feedback studies, quality assurance studies, and controlled interrupted time-series studies.

We identified studies that addressed AMU/ABU and AMR/ABR drivers at the mesolevel and microlevel only. Most of the studies focused on interventions, policies, and programs, which addressed AMU at mesolevel and microlevel only and AMR at macro-, meso-, and microlevels. Given the differences in the definitions of antimicrobials and antibiotics, we used the terms that were reported by the authors to ensure consistency with their findings. This means that there will be a mix of antimicrobial resistance/use and antibiotic resistance/use when describing the evidence. However, we found that the evidence is (almost) exclusively for antibiotic resistance/use as we did not find any substantial relevant evidence for other antimicrobials.

### 4.1. Drivers of Antibiotic Use

#### 4.1.1. Macrolevel Drivers

We identified no evidence addressing macrolevel drivers for antibiotic use.

#### 4.1.2. Mesolevel Drivers

The authors assessed the appropriateness of antimicrobial use in a paediatric critical care unit using a standardized assessment and found that the most common ways of inappropriately using antimicrobials were using overly broad-spectrum antibiotics, prescribing the wrong dosage, unnecessarily using antimicrobials when ineffective, and unwarranted overlap of spectrum [21]. Among physicians in a paediatric emergency department, there were significant variations in the choice of antimicrobials for management of pneumonia and low adherence to clinical practice guidelines [21, 22].

AMU, including the use of specific antibiotic classes and the prescribing of broad-spectrum antibiotics, varied across acute care hospitals. Notably, teaching hospitals had higher utilization rates [23]. Furthermore, respiratory and urinary tract infections were indicators of antibiotic utilization [24].

#### 4.1.3. Microlevel Drivers

Provider-level intrapersonal factors, such as fear of adverse outcomes and complications, fear of complaints and lawsuits, and reputational risk, influenced physician antibiotic prescription behavior [25]. Professional autonomy, benevolence, prior clinical experience and education, tolerance of uncertainty, overinterpretation of laboratory results, clinical presentation, and the perceived ability to communicate a decision were associated with increased antibiotic prescribing (high quality of evidence) [25–28]. Physician-patient interactions and physician perceptions may affect antibiotic prescriptions for respiratory tract infections—perception of patient desire for antibiotics was strongly associated with prescribing. However, patient desire for an antibiotic was not or was modestly associated with physician prescribing (4–13 stars for case-control studies using Newcastle-Ottawa scale and 22 points for cross-sectional studies using STROBE guidelines) [29]. Other interpersonal drivers including examples set by colleagues, social team dynamics, and hierarchies among physicians (i.e., difficulties in disagreeing/or advising a senior-level physician on antibiotic prescribing) also influenced physician prescribing behavior [25].

### 4.2. Drivers of Antibiotic Resistance

#### 4.2.1. Macrolevel Drivers

We identified no evidence addressing macrolevel drivers for antibiotic resistance.

#### 4.2.2. Mesolevel Drivers

The amount and timing of AMU may influence resistance rates and adverse outcomes among patients. For example, higher-use nursing homes, compared to lower-use nursing homes, had higher rates of adverse outcomes [30]. Among patients in long-term care facilities with pneumococcal disease receiving antibiotics, time since recent treatment (with the same antibiotic class), rather than prior cumulative exposure, was significantly associated with resistance [31]. A multicenter observational ecological study looking at antibiotic purchasing and hospital data found that, among 37 hospitals, hospital-specific use was *not* associated with decreased antibiotic susceptibility [32].

#### 4.2.3. Microlevel Drivers

Providers generally perceived the risk of AMR to be serious and optimizing the prescription process to be beneficial. Nevertheless, individual patient risks, patient expectations, and the belief that broad-spectrum antibiotics were effective and safer were barriers to conservative antibiotic prescribing [33].

Carbapenem, vancomycin and quinolone use, medical devices, ICU admission, underlying diseases, patient characteristics, and length of hospital stay were identified as the most significant risk factors for the presence of carbapenem-resistant *P. aeruginosa* [29].

### 4.3. Interventions That Aim to Manage Antibiotic Use

#### 4.3.1. Macrolevel Interventions

In Canada, the Choosing Wisely Canada Campaign, Public Health Ontario's Antibiotic Stewardship Strategy: Empiric Antibiotic Prescribing Guidelines, and the ASP campaigns implemented at the provincial level represent current national/provincial interventions/policies that aim at improving AMU. Policies and campaigns mainly included physician guidelines, guiding principles to develop guidelines, providing evidence-based tools, and patient pamphlets and videos [34–37]. United Kingdom developed a governmental toolkit known as TARGET (Treat Antibiotics Responsibly, Guidance, Education, Tools) to promote responsible use of antibiotics through resources for both physicians and patients [38].

#### 4.3.2. Mesolevel Interventions

ASPs may improve AMU, prescribing, costs, and length of hospital stay in various hospital settings. Significant decreases in consumption of antibacterial agents and antifungal agents, significant decreases in hospital costs, and improvements in patients' length of stay were seen after implementation of an ASP [39]. Similarly, in critical care settings, ASPs were associated with decreases in AMU, as well as lower antimicrobial costs per patient day and shorter durations of antibiotic therapy (poor quality of evidence). [40]. Likewise, the implementation of ASPs in paediatric hospital settings saw reductions in use and prescribing errors, without negative impacts on patient safety [41].

The duration and type of ASPs may also affect resistance rates. ASPs lasting longer than six months showed reductions in resistance. Interventions including formal antibiotic reassessment, deescalation protocol, computer-assisted decision support, or antibiotic practice guidelines were beneficial in improving use (poor quality of evidence) [40].

Rapid point-of-care tests were useful for diagnosing patients with influenza which may be helpful in guiding appropriate prescribing and deterring the inappropriate use of antibiotics for viral conditions (excellent-to-fair quality of evidence) [42]. We identified a negative relationship between the use of computer decisions support systems interventions and AMR. Such enablers may be effective at improving the adequacy of antibiotic coverage through guidance, which may also provide benefits to various patient outcomes, including resistance (poor quality of evidence) [43].

Use of, and adherence to, guidelines either implemented at high level or locally created de novo improved compliance to practice recommendations such as time to administration, prescription, and duration of antibiotics [44–47]. Légaré et al., Elligsen et al., and Steinberg et al. considered the utility of stewardship/training programs such as DECISION +2 training, the evaluation of prescribing patterns and recommendations by a stewardship team, and the general presence of ASP programs in Canadian ICUs [48–50]. All three studies showed improvements in decision-making behaviors of practitioners, decreased use of broad-spectrum antibiotics, and increased patient knowledge on appropriate use, respectively [48–50]. Implementation of communication interventions such as “systematic inter-professional pre-operative team briefing checklists” was associated with improved compliance to clinical practice guidelines on antibiotic administration [51]. Audit and feedback of AMU led to decreased monthly AMU directly following the intervention [52]. *Clostridium difficile* infection management policy resulted in an overall increase in treatment concordance, a reduction in the duration of AMU, and a decrease in hospital stay and mortality [53].

#### 4.3.3. Microlevel Interventions

Many antibiotic stewardship programs “fell short” of interventions including effective behavioral change techniques—self-monitoring, feedback, goal-setting, and action planning [54]. Furthermore, Semret et al. (2017) found that rapid multiplex testing may not be sufficient to reduce AMU. Moreover, radiographic suspicion, rather than rapid viral tests, appears to have a greater influence on physician antibiotic prescribing decisions [55].

Procalcitonin-guided therapy and C-reactive protein measurement reduced in the number of antibiotic prescriptions and use in the emergency department and the ICU (low risk of bias and low-to-moderate quality of evidence) [56–58]. For managing adults with acute respiratory tract infections, procalcitonin guidance reduced antibiotic prescriptions (low-to-moderate quality of evidence) [58] and lowered the risk of antibiotic-related side effects (high quality of evidence) [57]. However, in one study, the prescription reduction occurred only in the adult population (low risk of bias) [56]. Although C-reactive protein measurement can reduce prescription rates, it may also increase the number of patient return visits (low-to-moderate quality of evidence) [58]. The use of procalcitonin algorithms in the child and adult populations, as well as through audit and feedback interventions, resulted in notable cost-savings [59, 60]. Informational technology interventions and computerized decision support systems encouraged appropriate use of antimicrobials (poor quality and poor quality of evidence) [43, 61].

Other enablers and feedback mechanisms such as clinical surveillance software that identifies patients for prospective audit and feedback rounds resulted in significant reductions in broad-spectrum AMU and cost [62]. More broadly, enablers and restriction-type interventions were found to improve prescribing that aligned with hospital policies and decrease antibiotic treatment duration (high certainty of evidence) [63].

Clinician education on, and the implementation of, clinical practice guidelines reduced inappropriate management and increases in appropriate use (6-5 rating using the Newcastle-Ottawa Quality Assessment Tool) [27, 64]. Furthermore, both patient and clinician educational interventions—clinic or private setting education of patients/carers, public education campaigns, and combined and separated patient and clinician education—reduced antibiotic prescribing overall (low-to-moderate quality of evidence) [58]. Clinicians providing parents of children with delayed prescriptions for antibiotics significantly decreased antibiotic use, without reducing parent satisfaction (low quality of evidence) [65]. By contrast, delayed prescriptions may be limited by reductions in patient satisfaction (low-to-moderate quality of evidence) [58].

### 4.4. Interventions That Aim to Manage Antibiotic Resistance

#### 4.4.1. Macrolevel Interventions

Multimodal Infection Prevention and Control interventions implemented at national or subnational levels, instead of stand-alone interventions, demonstrated effectiveness (low-to-moderate quality of evidence) [66]. Others, including surveillance with active performance feedback, national hand-hygiene data for feedback at an individual level to drive behavioural change, care bundles and policies, development of guideline education, and training, were also effective (low-to-moderate quality of evidence) [66]. Hospitals in USA and Canada found that changing treatment regimens—for example, the treatment of *Clostridium difficile* infection with fidaxomicin as opposed to vancomycin—may limit the promotion of antibiotic-resistant bacteria, including vancomycin-resistant enterococci [67].

The WHO, the European Centre for Disease Control, the International Federation of Pharma Manufacturers and Associations, and the Centre for Disease Control and Prevention all developed approaches, guidelines, infographics, and frameworks to tackle AMR. Many aim at preventing the spread of AMR by providing guidance on Infection Prevention and Control practices, educational strategies, and action plans that can be adoptable/adaptable to specific contexts [2, 68–70]. Furthermore, the WHO developed a “global action plan” comprised of five objectives that are adaptable by member states to develop similar action plans [2].

#### 4.4.2. Mesolevel Interventions

Antibiotic stewardship objectives, implementation of guidelines, deescalation of therapy, switching from intravenous to oral treatment, therapeutic drug monitoring, the use of a list of restricted antibiotics, and bedside consultation supported improvements in adverse events and resistance rates (low quality of evidence) [71]. Multiple interventions, compared to single interventions, may reduce the rate of multidrug-resistant organisms in acute care hospitals [72]. On the other hand, the effectiveness of screening for endemic antibiotic-resistant organisms in hospital settings had little to no effect on antibiotic-resistant organism-related outcomes including incidence of infection, morbidity, mortality, and length of hospital stay [73].

While most of the identified systematic reviews (low-to-high quality of evidence) [74–77] tended to demonstrate that programs attempting to limit AMR had a positive impact, there was heterogeneity across studies [78]. A four-component program composed of ASP, standard care, environmental cleaning, and source control was most effective in reducing AMR (high-to-critical risk of bias) [76]. However, the effectiveness of these programs may vary depending on the specific bacterial infection assessed in hospital settings [77]. In contradiction, there is evidence that ASPs do not reduce antibiotic resistance in hospital settings [78]. Although there were differences in effectiveness that were also seen within studies—that is, comparing different hospital wards on reductions in health care-associated *Clostridium difficile* infections—adherence to ASP interventions in hospitals provided an overall benefit [74].

#### 4.4.3. Microlevel Interventions

Enablers, such as antimicrobial deescalation strategies, and rapid molecular testing coupled with communication interventions may be helpful in guiding appropriate use in hospitalized patients initially treated with broad-spectrum antibiotics (moderate quality of evidence) [79, 80]. Rapid molecular testing with direct communication of test results to clinicians or pharmacists significantly improved timelines to targeted therapy for a patient with bloodstream infections, when compared to standard rapid testing without any communication intervention. Rapid phenotypic techniques with direct communication could improve the timelines of targeted therapy [80]. Moreover, ICUs saw a protective effect on mortality with antimicrobial deescalation interventions (moderate quality of evidence) [79]. Pharmacists improved treatment-related and clinical outcomes and decreased costs. Pharmacist interventions posed no significantly negative impact relating to antimicrobial use within hospital settings (poor quality of evidence) [81]. A Canadian study found that viral testing was not associated with significant reductions in antibiotic use, odds of patient death, or length of hospital stay and resulted in more hospital resource utilization [82].

## 5. Discussion

This rapid review highlights that there are various in-hospital interventions that may address drivers of AMU/AMR. A significant number of studies focused on interventions (about 75%), while less (about 25%) focused on drivers. Recently, a shift in the understanding of what causes inappropriate use and AMR has paved a new understanding of this issue: there appears to be a wide range of sociocultural, context-specific, systems level, and historical factors that, together, contribute to this public health threat [15]. Our findings suggest that more research is needed in identifying these factors, particularly secondary and tertiary settings, at all three levels: macrolevel (national/regional systems and policies), mesolevel (health care organization and hospitals/hospital wards), and microlevel (clinician, patient/carer). Identifying these drivers may provide governments and healthcare systems with a formula for developing successful and targeted interventions and creating effective policies [15, 16].

At the macrolevel and mesolevel, hospital structures and hospital policies, such as the size of the hospital and guidelines, may influence antibiotic utilization and variations in management. Intra- and interpersonal microlevel factors impact inappropriate prescribing among clinicians. These include fear of adverse outcomes or litigation, the perceived ability to communicate a decision, physician-patient interactions, relationships with colleagues, and work dynamics.

Interestingly, we did not find studies that considered macrolevel drivers for both AMR and AMU. This may be due to the variations in the health care systems and governmental policies within OECD countries that make drivers of resistance and use at this level a challenging area to study. Studies in this area would provide insights into effective or ineffective health care systems and planning, which may influence resistance and help target potential flaws in the systems.

As for the hospital level (mesolevel), the amount and type of antibiotics used may affect resistance rates. Similar to AMU, misunderstandings among clinicians and patient expectations may impact antibiotic prescribing and, in turn, resistance rates.

Despite the lack of identified studies looking at macrolevel drivers for AMR, we did find one intervention study at this level. Infection prevention and control interventions, implemented on a national/subnational level, were effective at managing resistance. As for AMU, we did not find studies assessing interventions at the macrolevel. However, our grey literature search provided examples of national/international policies and interventions that have been implemented to improve both AMU and AMR.

Interventions that improve both AMU and resistance tended to be consistent across the mesolevel and microlevel, which were mainly comprised of implemented ASP, enablers (audit and feedback, deescalation protocols, and clinical practice guidelines), and training programs that modified clinician behaviors. For patients/carers, microlevel interventions, including clinician education, delayed prescriptions, and providing information, may improve AMU and decrease resistance rates.

### 5.1. Strengths and Limitations

This is the first evidence synthesis that illustrates the various drivers of inappropriate use and AMR at the macro-, meso-, and microlevels and interventions that may address these drivers in secondary and tertiary settings. The evidence was reviewed rapidly but systematically when possible.

To our knowledge, we provide an initial start to a health care systems framework categorizing the drivers and interventions at the macro-, meso-, and microlevels. Within a short timeframe, we employed rigorous systematic review methods to synthesize a large body of evidence with a few modifications to the review process. We conducted a comprehensive search and, in duplicate, we screened titles and abstracts and performed data abstraction. We recognized the differences between primary and secondary/tertiary care settings, as well as subsequent challenges influencing the drivers of, and interventions to address, misuse of antibiotics. With this in mind, we separated the evidence to elucidate the drivers and interventions that are specific in each setting. We worked closely with the Public Health Agency of Canada and crossed referenced our search results to ensure that we captured the relevant studies. Further, we did not pursue the same level of detail as established frameworks that assess social, cultural, and other contextual factors given our timelines to complete this rapid review.

We modified a few standard systematic review processes. Firstly, we did not conduct a risk of bias assessment and did not grade the overall certainty of the evidence of included studies. Secondly, instead of conducting full-text screening and data abstraction separately, we combined these two processes to meet timelines. Implementing these changes to the standard systematic review processes allowed us to meet rapid stakeholder deadlines.

While a systematic and comprehensive search strategy resulted in identifying many key publications, it is possible that some eligible material has been missed, given our modifications to the standard systematic review process. This may have put our review at higher risk of selection bias compared to a standard systematic review, leading to publication bias [83]. One solution for this is clear reporting and transparency for the methods used, which we have committed to. For future research, we recommend that other research groups conduct a standard systematic review to assess whether we have missed important information in this paper.

### 5.2. Implications for Practice and Research

Learned lessons from the COVID-19 pandemic, and the potential impact of COVID-19 policies on AMR, are an opportunity to better prepare for the long-term effects of AMR [84]. Countries are encouraged to refine their surveillance and data collection on the impacts of AMR to inform the future development of targeted policies and programs [84]. Targeted and coordinated actions can help manage the impact of AMR on our health, economy, and social inequality. A multifaceted approach is required which couples surveillance, infection, prevention, and control, stewardship, and research and development of new antibiotics and innovations [6].

Future studies should consider the structure of health care systems; macro-, meso-, and microlevels; and the interactions across and within levels when identifying the drivers of incorrect use of antibiotics and targeted interventions. We provide an initial start to a framework to help organize drivers and interventions at each level (Table 1).

## 6. Conclusion

This rapid evidence synthesis provides insight into the various drivers and interventions that may influence AMU and AMR among OECD countries. Most remarkably, we highlight drivers and interventions at various levels of the health care system (macro-, meso-, and microlevels) and demonstrate that one may influence the other—upstream drivers may lead to downstream events including inappropriate use and may, in turn, influence AMR. Considering these drivers and the complex relationships between health care networks and variations within secondary and tertiary settings, the implementation and design of future interventions or policies may be enhanced. Future research into drivers of AMR/AMU and the collection of data is essential to manage AMR and to build effective policies and interventions.

## Figures and Tables

**Table 1:** Framework stratifying drivers and interventions of AMR and AMU.

Level	Factors influencing AMR	Interventions, policies, and programs targeting AMR	Examples
*International, national, provincial* *Macrolevel*	Travel; trade; infrastructure; funding/health care expenditure; health insurance system; professional education; health literacy/awareness; health inequities/underserved groups	Regulation; legislation; fiscal measures; guidelines; communication/marketing; training; education	WHO global action plan on AMR; IDSA guidelines; drug coverage Ontario health insurance plan; awareness campaigns (i.e., *using antibiotics wisely* by choosing wisely Canada)
*Community, health network/sites* *Mesolevel*	Resources; health inequities/underserved groups, accessibility; affordability; social; culture; awareness	Regulation; communication/service provision; training; education	Quality indicators; management protocols; audit and feedback; educational campaigns (i.e., “*do bugs needs drugs?–a community program for wise use of antibiotics*”)
*Provider* *Microlevel*	Capability/experience; social opportunity (influences); physical opportunities (context, resources, time); reflective motivation; automatic motivation; communication with patient; liability; financial (dis)incentives	Training; education; enablers; restrictions; incentives; persuasion; examples/models	Reminders/alerts; decision aids; academic detailing
*Patient, caregiver* *Microlevel*	Knowledge/education; attitude; beliefs; behavior; communication with providers; socioeconomic status; access to care; health history; influence family/friends	Education; enablers (social support); restrictions; incentives; persuasion; examples/models	Educational materials; reminders/alerts; patient decision aids; adherence apps

## Data Availability

Underlying data can be requested by contacting the corresponding author.

## Supplementary Materials

Supplementary Materials Our summary of findings tables provide information on each article included in this review, such as the study outcomes, type of study, number of participants or studies included, certainty of the evidence, measure of effect, and main findings.
