# Supplementary material for: In-Hospital Macro-, Meso-, and Micro-Drivers and Interventions for Antibiotic Use and Resistance: A Rapid Evidence Synthesis of Data from Canada and Other OECD Countries
Source: Can J Infect Dis Med Microbiol. 2022 Mar 16;2022:5630361. doi: 10.1155/2022/5630361 (PMC9061047; doi:10.1155/2022/5630361)
Supplement: Supplementary Materials — Our summary of findings tables provide information on each article included in this review, such as the study outcomes, type of study, number of participants or studies included, certainty of the evidence, measure of effect, and main findings. [file 5630361.f1.pdf]

First Author (year)	Outcomes	Type of Study	Number of Participants/or number of studies included	Certainty of the evidence (high, moderate, low)	Measure of effect	Main findings	Comments (i.e. population)
Alak, A et al. (2010)	Compliance with antimicrobial prescriptions	Cross-sectional study	816 participants	Due to timelines, we did not assess the quality of the evidence for single studies.	N/A	Compliance with management guidelines was:<br>Children 5-18 years old: 59.7% (95% confidence interval (CI) 53%-66% n=211)<br>Children 3 months-5 years old: 83% (95% CI 80%-86%, n=605)<br>There were significant variations in the the antimicrobial used and non-recommened antimicrobials prescribed for children with pneumonia	Practitioner
Andrews, T. et al. (2012)	Antibiotic use	Systematic review	23 studies<br>The types of studies included:<br>Randomized control trials<br>Cluster randomized control trials<br>Non-reandomized control trials<br>Group pre/post-test	**Low quality**<br>Quality was assessed by the study using a framework adapted from the Cochran Handbook risk of bias tool	Odds ratio<br>Risk ratio	Physician consultation rates decreased by 13%–40% with written materials for both children and parents with a brief verbal education.<br>Video interventions moderately increased the proportion of parents with attitudes towards appropriate use of antibiotics (OR = 0.20, 95%CI: 0.10–0.39).<br>Delayed prescriptions reduced the number of reoported antibiotic use and did not reduce parent satisfaction (Risk Ratio = 0.46, 95% CI: 0.40–0.54).<br>The combined risk ration of five Interventions to influence antibiotic use in children with RTIs was RR=0.39 95% CI: 0.29–0.53.	Patient<br>Caregiver
Baur et al. (2017)	Change in the incidence of infection<br>Colonisation with antibiotic-resistant bacteria and *C di cile* infections	Systematic review & meta-analysis	76 qualitative studies<br>32 meta-analysis studies	**High quality**<br>There were some unexplained heterogeneity.	Incidence ratio	Antibiotic stewardship programmes reduced:<br>The incidence of infections and colonisation with multidrug-resistant Gram-negative bacteria (51% reduction; IR 0·49, 95% CI 0·35–0·68; p<0·0001)<br>Extended-spectrum β-lactamase- producing Gram-negative bacteria (48%; 0·52, 0·27–0·98; p=0·0428)<br>Meticillin-resistant *Staphylococcus aureus* (37%; 0·63, 0·45–0·88; p=0·0065)<br>The incidence of *C di cile* infections (32%; 0·68, 0·53–0·88; p=0·0029)	Clinician
Baysari et al. (2016)	Appropriate use of antimicrobials<br>Patient mortality<br>Hospital length of stay (LOS)	Systematic review & meta-analysis	45 studies	**Poor**<br>The quality was assessed using a 10-point rating scale previously used to assess studies reporting evaluations of computerized decision support system.	Relative risk	Appropriateness: IT interventions were associated with an increase in appropriate use of antimicrobials based on the random effects model (pooled RR from DerSimonian-Laird method: 1.49, 95%CI: 1.23–1.81, p < 0.0001; pooled RR from Knapp-Hartung adjustment: 1.49, 95%CI: 1.07–2.08, p < 0.05). Mortality: there was no impact on mortality based on the fixed effect model (pooled RR: 0.91, 95%CI: 0.82–1.00, p = 0.06). Hospital LOS: there was no association between introduction of an IT intervention for antimicrobials and LOS (pooled mean difference: −0.84, 95%CI: −2.43 to 0.76, p = 0.30).	Clinician<br>Patient
Bertollo et al. (2018)	Antibiotic resistance	Systematic reviews	26 studies	The authors did not assess the quality of the evidence.	N/A	No evidence that Antibitoic Stewardship Programs are effective in reducing antibiotic resistance in hospital settings.	Clinician
Bond et al. (2014)	Primary outcome: Effectiveness of screening for endemic antibiotic resistant organisms	Summary of:<br>Systematic reviews<br>Primary studies<br>Evidence-based guideline	7 systematic reviews<br>6 primary studies<br>5 clinical practice guidelines	Quality was narratively described by one author	Intervention screening for endemic antibitoic resistant organisms	1. Systematic review findings<br>Higher quality individual studies (RCTs and reliable quasi-experimental studies) show that screening has little to no effect on antibiotic resistant organisms-related outcomes. There was no evidence regarding health care associated antibiotic resistant organism-related morbidities and mortality.<br><br>2. Primary studies<br>No studies examined the effectiveness of universal screening vs. no screening. The results of a single, large cluster randomized trial strongly suggest that, in the short term, screening and isolation and screening and targeted decolonization are not as effective as a protocol of no screening and universal decolonization in reducing MRSA colonization and infection in ICU patients.<br><br>Given that studies included other interventions in addition to screening, it was difficult to conclude that the reduction of antibiotic resistant organisms was due to screening.<br><br>3. Clinical guidelines<br>Majority of guidelines recommended admission screening of high-risk patients in endemic settings. No guideline recommended a universal screening strategy for selected anitbitoic resistant organisms nor did any recommend routine staff screening of antibitoic resistant organisms.	N/A
Buehler SS. Et al. (2016)	The effectiveness of three rapid diagnostic practices in decreasing the time to targeted therapy for hospitalized patients with bloodstrem infections	Systematic review	16 studies included<br>5140 study participants	The study did not conduct a quality assessment.	N/A	Rapid molecular testing with direct communication significantly improves timeliness compared to standard testing.<br>Rapid phenotypic techniques with direct communication likely improve the timeliness of targeted therapy.<br>Studies show a significant and homogeneous reduction in mortality associated with rapid molecular testing combined with direct communication.<br>Despite there being no firm recommendation, the data do suggest that each of these three practices has the potential to improve times to initiate targeted therapy and possibly improve other patient outcomes.	Detection<br>Provider
Curtis et al. (2017)	Adequacy of antibiotic coverage<br>Mortality<br>Volume of antibiotic usage<br>Length of stay<br>Antibiotic cost<br>Compliance with guidelines<br>Antimicrobial resistance<br>CDS implementation and uptake	Systeamtic review & meta-analysis	81 studies	**Poor quality**<br>The quality was assessed a 10-point rating scale	Odds ratio	Computerized Decision Support significantly improved the adequacy of antibiotic coverage.	Clinician
Davey et al. (2015)	To update a systematic review assessing the application of Behaviour Change Techniques (BCTs) to improving hospital antibiotic prescribing:<br>- self-monitoring<br>- feedback<br>- goal setting<br>- action planning.	Systematic review of randomised or controlled clinical trials<br>Controlled before and after studies<br>Interrupted time series	116 studies	The authors did not assess the quality of the evidence.	N/A	Reporting of BCTs was poor, with little detail of BCT characteristics, feedback was only reported for 17 (13.8%) of the interventions and 3 (2.4%) included action planning, and self-monitoring was used in only 1(0.8%) intervention. Goals were reported for all interventions but were poorly specified, with only three of the nine characteristics reported for ≥50% of interventions. A goal threshold and timescale were specified for just 1 of the 123 interventions.	Provider
Davey et al. (2017)	Antibiotic prescription	Systematic review	221 studies:<br>58 randomized control trials<br>163 non-randomized studies	**High certainty evidence**<br>The quality was assessed using Grading of Recommendations, Assessment, Development and Evaluation (GRADE)	Risk difference	The purpose was to estimate the effectiveness and safety of interventions to improve antibiotic prescribing to and to investigate the effect of restriction and enablement.<br>Hospital inpatients were treated according to antibiotic prescribing policy with the intervention compared with no intervention [RD 15%, 95% confidence interval CI: 14% to 16%]<br>Antitbiotic duration decreased from 11.0 to 1.95 days<br>Restriction/enablement showed assocations with improving prescribing according to polices, and were independenly asssociated with increased compliance with policies.<br>Enablement enhanced restrictive interventions, and feedback was "probably" more effective among enabling interventions.	Provider
Didiodato et al. (2016)	Incidence of healthcare-associated Clostridium difficile infection	Observational study	339 beds	Due to timelines, we did not assess the quality of the evidence for single studies.	Rate ratio	A daily prospective audit and feedback resulted in a significant reduction in the risk of health care associated Clostridium difficile infection on the medicine wards, independent of an overall reduction in antibiotic utilization. The ward-level burden of Clostridium difficile was shown to significantly increase the risk of health care associated Clostridium difficile.	Clinician
Doyon, S. et al. (2009)	Guideline and prescription compliance for acute community-acquired pneumonia management in a paediatric university hospital centre before and after its dissemination	Uncontrolled quasi-experimental study	1151 prescriptions:<br>439 pre-intervention cases<br>339 post-intervention cases	Due to timelines, we did not assess the quality of the evidence for single studies.	Odds ratio<br>Event count	Guideline dissemination intervention: first, networking and consultation with either a paediatric emergency doctor peer leader by email (emergency department) or through consultations in a small group with a paediatrician peer leader (wards). Guideline dissemination included mailing and emailing official guidelines, sending out a prescription pre-printed sheet, organizing an information meeting led by a paediatric peer leader for residents and further networking. Guideline dissemination involved the participation of prescribers at every step of the process, except mailing and emailing, which affirmed the active and multiple nature of the intervention.<br><br>Results: guideline dissemination significantly increased prescription compliance. The prescription compliance with the guidelines increased from 20.1% in the pre-intervention period to 52.9% in the intervention period. Inappropriate antimicrobial choice represented 66.6% of the causes of non-compliance in the pre-intervention period and 42.1% in the intervention period.	Practitioner<br>Tertiary care pediatric hospital
Egilmezer et al. (2018)	Antiviral prescription<br>Antibiotic prescription<br>Patient length of stay in the ED	Systematic review	30 studies	**Nine studies were graded level 2 or excellent quality, 12 were graded level 3 or good quality, nine were graded level 4 or fair quality**<br>The authors assessed quality using the Oxford Centre for Evidence based Medicine (OCEBM) guidelines	N/A	A positive point-of-care testing result significantly increased use of antivirals for influenza compared with negative result and standard supportive care. Positive point-of-care tests also led to a decrease in antibiotic use.	Clinician<br>Patient
Flokas et al. (2017)	Inappropriate antibiotic treatment	Systematic review	8 studies:<br>Prospective cohort<br>Retrospective cohort	Majority of studies were given a rating 6, and lowest was a rating of 5. Quality was judged based on Newcastle–Ottawa Quality Assessment Tool with a maximum score of 6. The study did not provide an overall rating of the quality of the studies.	N/A	Education on clinical practice guidelines, asymptomatic bacterium, and differentiating between cases of asymptomatic bacterium and urinary tract infections resulted in up to 80% reductions in inappropriate management of asymptomatic bacterium.	Practitioners

Légare et al. (2013)	Antibiotic prescribing behaviour for:<br>Physicians receiving the DECISION+2 training<br>Physicians that did not received DECISION+2 training	Multi-center, two- arm, parallel randomized clustered trial	9 physicians	Due to timelines, we did not assess the quality of the evidence for single studies.	Event count	DECISION + 2 positively influenced shared decision making behaviors as assessed by patients and teacher physicians. Physicians' intention to engage in SDM was not affected by DECISION + 2.	Student physicians<br>Teaching hospital setting
Gauthier, M. et al. (2009)	Overall appropriateness of ceftriaxone use	Before and after controlled study	32 ED physicians<br>127 ED patients	Due to timelines, we did not assess the quality of the evidence for single studies.	Odds ratio	Overall, after the implementation of the guideline, the patients were twice as likely to receive adequate treatment with ceftriaxone (crude OR, 2.2; 95% CI, 0.5Y9.0); this finding was not, however, statistically significant. Indications for prescription of ceftriaxone were adequate in 16.7% of the preguideline and 22.4% of the post guideline groups (P = 0.4). Physicians were twice as likely to use ceftriaxone adequately after the guideline's implementation, but this result was not statistically significant (crude odds ratio, 2.2; 95% confidence interval, 0.5Y9.0).	Practitioner (provider)<br>Patient (parents of children receiving care)
Hurford et al. (2012)	Effectiveness of model to assess atribubtes of:<br>Antimicrobial stewardship program<br>Antibiotic prescription	Components of an antimicrobial stewardship program were to alter the fraction of patients prescribed antimicrobials, to shorten the average duration of treatment, and to alter the types of antimicrobials prescribed. These components were incorporated into a mathematical model that was compared to data reporting the number of patients colonized with Pseudomonas aeruginosa and the number of patients colonized with antimicrobial-resistant P. aeruginosa first isolates before and after the antimicrobial stewardship program	N/A	Due to timelines, we did not assess the quality of the evidence for single studies.	N/A	Analysis shows that the reported decrease in the number of patients colonized was due to treating fewer patients, while the reported decrease in the number of patients colonized with resistant P. aeruginosa was due to the combined effect of treating fewer patients and altering the types of antimicrobials prescribed.<br>It also finds that shortening the average duration of treatment was unlikely to have produced any noticeable effects and that further reducing the fraction of patients prescribed antimicrobials would most substantially reduce P. aeruginosa antimicrobial resistance in the future.<br>The analytical framework considers the effect of colonization pressure on infection spread and can be used to interpret clinical antimicrobial resistance data to assess different aspects of antimicrobial stewardship within the ecological context of the intensive care unit.	Mount Sinai Hospital, Toronto
Kaki et al. (2011)	Efficacy of antimicrobial stewardship program on:<br>Antibiotic use<br>Costs<br>Appropriateness of antibiotic<br>Duration of therapy/timing<br>Rates of antibiotic resistant clinical outcomes (length-of-stay, hospital-acquired infections, mortality)	Systematic review	24 studies:<br>3 randomized control trials<br>18 uncontrolled before and after study<br>3 interrupted time series<br>42,223 participants	**Poor quality**<br>(Cochrane Effective Practice and Organization of Care (EPOC) Review Group inclusion criteria for randomized controlled trials, interrupted time series and controlled before-and-after studies)	N/A	Antimicrobial stewardship led to:<br>1. Reducitions in antibiotic use by 11%–38% defined daily doses/1000 patient-days<br>2. Lower antibiotic costs by US$ 5–10/ patient-day<br>3. Less inappropriate use<br>Shorter average duration of antibiotic therapy<br>4. Beneficial effect on institutional antibitoic resistance with a formal antibiotic reassessment, de-escalation protocol, computer-assisted decision support or antibiotic practice guidelines<br><br>Stewardship interventions beyond 6 months were associated with reductions in antimicrobial resistance rates, although this differed by drug–pathogen combination<br><br>There were no significant difference in the frequency of hospital-acquired infections between periods with and without antimicrobial stewardship, most studies documented no significant difference on the length of stay, no studies detected a significant increase in overall intensive care mortality.	N/A
Karanika et al. (2016)	Efficacy in terms of antimicrobial consumption before and after the implementation of an ASP in hospitals<br>Effect of an ASP on a series of clinical outcomes, including measurement of antimicrobial consumption with high resistance potential (defined as the antimicrobials whose resistance occurs during drug development or clinical trials, or within 2 years of general use, such as carbapenems and glycopeptides), overall and infection-related 30-day mortality, length of stay in hospital (LoS), and intensive care unit (ICU) stay, change in Clostridium difficile infection rate, change in rates of resistant strains throughout the hospitals, total infection rate, and consistency of antimicrobial treatment with ASP or national guidelines, as well as the change on the cost of antimicrobial treatment.	Systematic review & meta-analysis	26 studies	The methodological quality was assessed using the measurement tool Newcastle Ottawa scale (NOS). The study did not provide an overall rating of the quality of the studies.	Overall decrease in consumption (by percentage)<br>Risk Difference for decrease in infections.	After implementation of an antibiotic stewardship program, the authors found:<br>A decrease in antimicrobial consumption of 19.1% (95% CI [30.1 to 7.5])<br>Six studies showing a decrease in consumption of antifungal medicines by 39.1% (95% CI [62.3 to 16.0])<br>A decrease in consumption of strictly antimicrobial agents by 26.6% (95% CI 52.3 to 0.8)	Provider behaviour
Leache et al. (2018)	Treatment-related outcomes (TROs),<br>Clinical outcomes (COs),<br>Cost and microbiological outcomes (MOs).	Systematic review	23 studies	**Poor quality**<br>The quality was assessed using the Cochrane risk of bias tool	N/A	Pharmacists improve TROs and COs, and decrease costs.	Clinician<br>Patient
Lee A. et al. (2018)	Impact of a clinical surveillance software used to identify patients for prospective audit and feedback rounds	Before and after/implementation study	552 beds	Due to timelines, we did not assess the quality of the evidence for single studies.	N/A	Results from a general internal medicine unit show statistically significant reductions in the use of broad-spectrum antibiotics and antibiotic expenditures, with no untoward changes in key clinical and patient safety outcomes.	Patient
Lee, C. et al. (2015)	Prevalence of antimicrobial utilization	Cross-sectional study	1021 patients:<br>508 acute care<br>513 long term care	Due to timelines, we did not assess the quality of the evidence for single studies.	N/A	One or more antimicrobial agents were ordered in 31% and 4% of acute care and long-term care patients, respectively.<br><br>Respiratory and urinary tract infections were the most common indication for antimicrobial therapy in both acute and long term care.<br><br>About 25% of surgical prophylaxis orders were prescribed for greater than 24-hours.	Patient
Lee, T. C. et al. (2014)	To optimize antibiotic use through trainee-led time-outs.	Before and after study	417-bed tertiary care hospital<br>679 participants	Due to timelines, we did not assess the quality of the evidence for single studies.	N/A	Total costs in the units decreased from $149 743CAD (January 2011 to January 2012) to $80 319 (January 2012 to January 2013), for a savings of $69 424 (46% reduction).<br><br>Of the savings, $54 150 (78%) was related to carbapenems and $15 274 (22%) was due to other antibiotic classes.<br><br>Adherence with the auditing process was 80%. In the time-series analyses, the only reliable and statistically significant change was a reduction in the rate of moxifloxicin use, by −1.9 DDDs per 1000 patient-days per month (95% CI, −3.8 to −0.02; P = 0.048).<br><br>Rates of C. difficile infection decreased from 24.2 to 19.6 per 10 000 patient-days (incidence rate ratio, 0.8 [CI, 0.5 to 1.3]).	Patients
Leung et al. (2011)	Cost and utilization of antimicrobials<br>The rate of nosocomially acquired *C. difficile* infection	Prospective audit and feedback, followed by daily discussion for optimization of therapy (pilot period) compared to previous year	12 beds (1127 patient-days)	Due to timelines, we did not assess the quality of the evidence for single studies.	Defined daily doses (DDD), absolute reductions	The average antimicrobial costs decreased significantly after implementa-tion of antimicrobial stewardship (*p* = 0.024). absolute reduction of 24.57 DDDs/100 patient-days (38.9%).	Clinician
Lingard et al (2011)	Timing of preoperative antibiotic administration as compared to accepted treatment guidelines	Retrospective pre-intervention/ post-intervention study design	680 participants:<br>340 pre-intervention<br>340 post-intervention	Due to timelines, we did not assess the quality of the evidence for single studies.	N/A	There was a lot of variability in medicial chart documentation practices, both between study sites and between individual practitioners and this caused some difficulities to analyze the data.<br>.<br>In cases where the timing of antibiotics administration was documented unambiguously in the chart, antibiotic prophylaxis was on time for 77.6% of cases in the pre-intervention phase of the study, and for 87.6% of cases in the post-intervention phase (p<0.01).<br><br>For every level of antibiotic untimeliness (clearly-not-on- time, clearly-problematic and potentially-problematic), there was no statistically significant effect of the timing of the team briefing on antibiotic timeliness.	Patient
Francis et al. (2010)	Median time to antibiotic administration in the emergency department in severe sepsis patients before and after the implementation of an ED sepsis protocol<br><br>Compliance with sepsis guidlinesfor time to antibiotics in patients with severe sepsis.	Cross-sectional study	213 patients	Due to timelines, we did not assess the quality of the evidence for single studies.	Event count	Administration of an ED sepsis protocol significantly improves the time to administration of antibiotics as well as the appropriateness of the initial antibiotic therapy in patients with severe sepsis.	Health care professionals in the ED
Martin et al. (2015)	Adherence to guidelines in the management of Staphylococcus aureus bacteremia, as outlined in the internal policy	Before and after study	128 Staphylococcus aureus bacteremia cases between 2011 and 124 cases in 2012	Due to timelines, we did not assess the quality of the evidence for single studies.	N/A	In patients who survived the minimum treatment course (greater than 13 days), there was a significant improvement in adherence to IDSA guidelines in 2012.<br><br>In terms of choice of antibiotics, the proportion of patients who did not receive empiric vancomycin in addition to cefazolin or cloxacillin decreased significantly between 2011 and 2012.	Patient<br>Tertiary care teaching hospitals, Hamilton, Canada.

McDonagh et al. (2018)	Summarized and updated a previously performed systematic review of interventions to reduce inappropriate use of antibiotics for acute RTIs	Updated systematic review	13 studies<br>8 RCTs<br>5 Observational<br>7,452,357 patients or parents<br>101,443 clinics or clinicians	**Low to moderate quality**<br>All articles were appraised using the Effective Public Health Practice Project tool	Relative risk	Parent education interventions had a 21% reduction in antibiotics prescribed for acute respiratory tract infections (ARTIs).<br>Combined patient/clinician education interventions showed a 7% reduction in antibiotics prescribed for ARTIs.<br>Procalcitonin point-of-care testing for adults with RTIs found a 12% to 72% reduction in the antibiotics prescribed.<br>Electronic decision support systems showed a 24% to 47% improving in antimicrobial prescribing practices, as well as a 5% to 9% reduction in the amount of antimicrobials prescribed.<br>All of these interventions had no increased complications, no adverse consequences, no changes in patient complication and satisfaction, and there was also no increases in patients returning for a visit.<br>Interventions that reduced and delayed prescribing overall prescribing but some limitations including longer duration of symptom and decreased patient satisfaction.<br>C-reactive protein (CRP) measurement: 13% to 33% reduction in antibiotic prescribing. But there were increases in return visits (RR=1.64, 95% CI 1.35-2.00).<br>Multifaceted interventions: clinician communication + CRP measurement led to 70% reduction in prescribing (OR = 0.30 95%CI=0.26-0.36).<br>Increase in hospitalizations at 1 month.<br>Educational interventions that target patients/parents had higher reductions in prescribing. Combined patient and clinician educational interventions did not see greater reductions.	Patients<br>Providers<br>Community
Mulpuru et al. (2015)	Number of inpatient deaths<br>Length of hospital stay<br>Admission to the intensive care unit (ICU)<br>Measures of resource utilization (antibiotic and antiviral prescriptions, chest radiograph and computed tomography imaging, blood and sputum cultures, bronchoscopy)<br>Use and duration of isolation precautions in the hospital	Retrospective observational cohort	24,567 hospital admissions (7,327 unique patients)	Due to timelines, we did not assess the quality of the evidence for single studies.	Odds ratio	Viral testing was performed in 11% (2,722/24,567) of hospital admissions and was not associated with reduced odds for death (odds ratio 0.90, 95% CI 0.76–1.10) or longer length of stay (+1 day for those tested).<br><br>Viral testing resulted in more resource utilization, including intensive care unit admission,but positive test results were not associated with less antibiotic use or shorter duration of isolation.	Patient
Nerandzic et al. (2012)	Comparison of incidence of vancomycin-resistant enterococci (VRE) and Candida species acquisition–AMR/resistant organisms	Double-blind, randomized, phase III clinical trial	548 participants:<br>265 treated with fidaxomicin<br>283 treated with vancomycin	Due to timelines, we did not assess the quality of the evidence for single studies.	N/A	In comparison with vancomycin-treated patients, fidaxomicin- treated patients had reduced acquisition of VRE (7% vs 31%, respectively; P < .001) and Candida species (19% vs 29%, respectively; P = .03).<br>For patients with preexisting VRE, the mean concentration of VRE decreased significantly in the fidaxomicin group but not the vancomycin group. Most VRE isolates recovered after fidaxomicin treatment had elevated fidaxomicin minimum inhibitory concentrations, and subpopulations of VRE with elevated fidaxomicin MICs were common before therapy. Fidaxomicin was less likely than vancomycin to promote acquisition of VRE and Candida species during CDI treatment. However, selection of preexisting subpopulations of VRE with elevated fidaxomicin minimum inhibitory concentrations was common during fidaxomicin therapy.	Patient
Popovski et al. (2015)	The implementation of the internal guideline would result in a shift from fluoroquinolone to cephalosporin use and in a reduction in the treatment duration	Implementation study	152 and 145 patients with intra-abdominal infections were included in the pre- and post-intervention periods	Due to timelines, we did not assess the quality of the evidence for single studies.	N/A	There was a significant reduction in the proportion of patients who received ciprofloxacin therapy from 74% to 34% (OR 0.18, 95% CI 0.11–0.31) and in DOT/1000 PD from 221 to 74 (OR 0.3, 95% CI 0.2–0.3). Also, a reduction in the DOT/1000 PD for piperacillin/tazobactam was seen (from 116 to 67; OR 0.6, 95% CI 0.5–0.7). There was an increase in the use of ceftriaxone from 1.3% to 53% of patients (OR 85, 95% CI 20–515) and from 6 to 92 DOT/1000 PD (OR 17, 95% CI 10–25). This change in practice was sustained over .2 years since the end of the active intervention, as shown in the unit-wide antimicrobial utilization data.	Patient
Price et al. (2018)	Effectiveness of infection prevention control (IPC) programs interventions implemented at national or subnational levels to inform the development of WHO guidelines on the core components of national IPC programmes.	Systematic review	29 studies:<br>9 interrupted time-series studies<br>9 cluster-randomized trials<br>5 controlled before-and-after studies<br>1 non-randomized trial<br>5 economic evaluations)<br>No reported number of participants.	**Low to moderate quality**<br>Quality was assessed using:<br>1. The Effective Practice and Organisation of Care (EPOC) risk-of-bias criteria<br>2. The Grading of Recommendation, Assessment, Development, and Evaluation (GRADE)<br>3. The Phillips' checklist 21	N/A	Not directly about antimicrobial mis/overuse. Talks about interventions including: multimodal, care bundles, policies, surveillance, monitoring, and feedback.<br><br>With multimodal intervention, majority of studies showed effectiveness of reducing infections.<br><br>Care bundles and polices were shown to be effective but studies were subject to bias and low quality.	
Rawson et al. (2017)	Hospital unit level<br>Patient level<br>Prescriber level outcomes	Systematic review	58 studies:<br>18 primary care<br>40 secondary care	**Low to medium for majority of clinical outcomes, and high-quality evdience supporting clinical decision support systems at the unit level and prescriber level**<br>The quality was assessed using the Criteria for the Review Of Multiple Study designs (ICROMS) criteria	N/A	Greater consideration of the factors that drive non-expert decision making must be considered when designing clinical decision support system interventions.	Clinician<br>Patient
Schuetz et al. (2017)	Safety and efficacy of using procalcitonin for starting or stopping antibiotics over a large range of patients with varying severity of ARIs and from different clinical settings	Systematic review	26 randomized control trials<br>6708 participants	**High quality**<br>The study used Grading of Recommendations, Assessment, Development, Evaluation (GRADE)	Odds ratio	Procalcitonin (biomarker) that is used to guide appropriate use of antibiotics for acute respiratory tract infections was associated with a 2.4-day reduction in antibiotic exposure (5.7 versus 8.1 days, 95% CI -2.71 to - 2.15, P < 0.001), and resulted in lower risk of antibitoic-related side effects (16.3% versus 22.1%, adjusted OR 0.68, 95% CI 0.57 to 0.82, P < 0.001).	ED<br>ICU
Schuts et al. (2016)	Antibitoic stewardship program effect on:<br>Clinical outcomes<br>Adverse events<br>Costs<br>Bacterial resistance rates	Systeamtic review & meta-analysis	145 studies<br>21,945 participants	**Generally low quality, and heterogeneity between studies was moderate to high**<br>The study used:<br>1. Cochrane Risk of Bias tool for RCTs<br>2. The Quality in Prognostic Studies tool for prognostic factors<br>3. Adapted version of the Newcastle-Ottawa Quality Assessment Scale for non-RCTs	Relative risk	Use of empirical therapy according to guidelines, de-escalation of therapy, switch from intravenous to oral therapy, therapeutic drug monitoring, use of a list of restricted antibiotics, and bedside consultation can lead to significant benefits for clinical outcomes, adverse events, and costs, although the quality of evidence is generally low. Guideline-adherent empirical therapy was associated with a relative risk reduction for mortality of 35% (relative risk 0·65, 95% CI 0·54–0·80, p<0 0001) and for de-escalation of 56% (0·44, 0·30–0·66, p<0 0001).	Provider behaviour
Semret et al. (2017)	Impact of rapid viral test results on:<br>Antimicrobial prescriptions<br>Clinical outcomes among hospitalized adult	Prospective observational study using secondary data	800 participants	Due to timelines, we did not assess the quality of the evidence for single studies.	N/A	The influenza virus was a major cause of adult hospital admission for respiratory tract infections. Despite this, the study found that access to rapid multiplex testing may not be sufficient to reduce antibiotic use. Rather, clinicans seemed to make antibiotic decisions based on radiographic findings rather than virual test results, likely due to concerns over bacterial infection. Two retrospective analyses from Canada showed that influenza virus positivity led to increased use of antivirals, but test results did not correlate with overall antibiotic use.	Provider
Smith et al. (2015)	Decreases in antimicrobial utilization<br>Prescribing errors<br>Drug costs	Systematic review of:<br>Cohort<br>Before and after studies<br>Cross-sectional<br>Quasi-experimental studies	10 studies	The authors did not assess the quality of the evidence.	N/A	These studies demonstrate reductions in antimicrobial utilization, cost, and prescribing errors with no apparent negative impact on patient safety. The study found decreases in antimicrobial utilization (11 studies), prescribing errors (3 studies), and drug costs (3 studies) with antibiotic stewardship programs. Five studies observed no adverse effects of antibitoic stewardship interventions on patient safety. Data to support an association between pediatric antibiotic stewardship interventions and antimicrobial resistance are limited.	N/A
Steinberg et al. (2016)	Presence of an antimicrobial stewardship at the physician's institution	Cross-sectional study	634 Canadian physicians screened<br>185 physicians completed the survey.	Due to timelines, we did not assess the quality of the evidence for single studies.	N/A	74% of respondents reported that there was at least 1 component of an antibiotic stewardship program at their institution.<br><br>86% of respondents agreed or strongly agreed that the patients in their ICU benefit from an antibiotic stewardship program, with 81% reporting the programs increases their knowledge of appropriate antimicrobial use in the ICU setting.<br><br>Only 11% of respondents reported they felt that interacting with the antibiotic stewardship program team was an inefficient use of their time, and only 7% expressed concern that the antibiotic stewardship program impacted their autonomy.<br><br>These components included infectious diseases (ID) physician and/or ID pharmacist rounding with ICU team (40%, n =73), audit and feedback for all antimicrobials prescribed within the ICU (14%, n = 25), audit and feedback for certain antimicrobials<br><br>Antibitoics prescribed within the ICU was 21%, antimicrobials restricted to ID consultants was 40%, and antimicrobials restricted to staff ICU physicians was 26%.	Clinician
Tabah et al. (2016)	Efficacy of antimicrobial de-escalation on duration of antimicrobial therapy<br>Emergence of resistance<br>Emergence of costs<br>Mortality	Systematic review of:<br>Randomised controlled trials<br>non-randomized control trials	14 studies:<br>2 randomized control trials<br>12 cohort studies<br>2462 participants	**Moderate quality**<br>The quality was appraised using:<br>1. The Cochrane Risk of Bias tool for randomized controlled trials<br>2. A modified version of the Newcastle-Ottawa Scale for case-control and cohort studies	Relative risk	Antimicrobial de-escalation did not reduce the total duration of antimicrobial treatment costs or length of stay.<br>The effects of antimicrobial de-escalation on bacterial resistance have not been adequately investigated.<br>Antimicrobial de-escalation may be protective against mortality but there is too bias to make conclusions.	N/A
Taggart et al. (2015)	Total systemic (oral or parenteral) antimicrobial use in each ICU<br>Use of pre-specified antibiotic agents or classes<br>Antimicrobial costs<br>Antimicrobial susceptibility for Escherichia coli and Pseudomonas aeruginosa, Clostridium difficile infection incidence<br>clinical outcomes (monthly ICU mortality rates, ICU length of stay and 48-hour ICU re-admission rates)	Controlled interrupted time series study	4 adult ICUs included:<br>19-bed trauma and neurosurgery ICU (TNICU)<br>24-bed medical and surgical ICU (MSICU)<br>The control ICUs included:<br>15-bed cardiovascular surgery ICU (CVICU)<br>10-bed cardiac ICU (CICU)	Due to timelines, we did not assess the quality of the evidence for single studies.	Mean total monthly antimicrobial use in defined daily doses (DDD) per 1000 patient days	Mean total monthly antimicrobial use in defined daily doses (DDD) per 1000 patient days was reduced by 28% in the TNICU but increased by 14% in the MSICU<br>In the time series analysis, total monthly antimicrobial use in the TNICU decreased by 375 DDD per 1000 patient days (p < 0.0009) immediately following the intervention, followed by a non-significant downward trend in use of −9 DDD per 1000 patient days (p = 0.56).	Clinician

Teerawattanapong et al. (2017)	Multi-drug resistant Gram negative bacilli acquisition<br>Multi-drug resistant Gram negative bacilli colonization<br>Multi-drug resistant Gram negative bacilli infection	Systematic review & network meta-analysis	42 studies	**High for each included randomized control trial, 34 observational studies were rated as moderate risk of bias, 2 observational studies as serious risk of bias,and 1 study as critical risk of bias**<br>The Cochrane Collaboration's risk of bias and ROBINS-I tools were used to assess the quality of the evidence	Rate ratio	Compared with standard care, a 4-component strategy composed of standard care, antimicrobial stewardship program, environmental cleaning, and source control was the most effective intervention (rate ratio, 0.05 [95% con dence interval, .01–.38]).	Clinician
van der Does et al. (2016)	Antibiotic prescription	Systematic review	9 randomized control trials	**Low risk of bias**<br>The authors used QUADAS-2 was used to assess risk of bias	N/A	Procalcitonin guided therapy significantly reduced the number of antibiotic prescriptions in adults, but did not reduce the number of prescriptions issued for children.	ED physician
Voor et al. (2014)	Effective infection prevention strategies related to hospital microorganisms<br>Environmental sources and reservoirs	Systematic review	162 studies	**Case-control studies scored between 4 and 6 stars of 10 stars with the Newcastle-Ottawa scale, and cohort studies scored between 6 and 7 stars of 13 stars. Cross-sectional studies and the single study with an observational study design, all according to STROBE guidelines, resulted in scores of 15, 17, and 18 points of a total of 22**<br>The quality was assessed using the STROBE guidelines for included cross-sectional studies as well as the Newcastle-Ottawa quality assessment scale for included case-control and cohort studies	N/A	The use of barrier and/or contact precautions was found to be the most successful intervention strategy,followed by patient cohorting and active surveillance, enhanced hand hygiene, enhanced environmental cleaning, staff educational programs, staff cohorting, equipment cohorting/single-use equipment.	N/A
Wang et al. (2017)	The authors evaluated the association between antibiotic utilization and resistance in academic and community hospitals in Ontario, Canada<br>Antibiotic consumption was defined as daily doses per 1,000 patient days (DDDs/1,000 PD).	Observational ecological study	37 hospitals	Due to timelines, we did not assess the quality of the evidence for single studies.	N/A	The autors found that increased antibiotic consumption was associated with decreased antibiotic susceptibility for Pseudomonas aeruginosa. However, increased antibiotic consumption predicted increased antibiotic susceptibility significantly for scherichia coli, Enterobacter spp, and Enterococcus spp, and nonsignificantly for Staphylococcus aureus.<br><br>The type of hospital (medium community hospital vs. large community hospital vs. teaching hospitals) and laboratory standard did not significantly predict antibiotic susceptibility.<br><br>Increases in patient stay in the hospital predicted increased organism susceptibility.	Medium community hospitals<br>Large community hospitals<br>Teaching hospitals
Westwood et al. (2015)	Clinical effectiveness<br>Cost-effectiveness	Systematic review	18 randomized control trials studies	**"Unclear quality due to limitation in reporting"**<br>The quality was assessed using the Cochrane Risk of Bias tool	Relative risk	In the ICU, Procalcitonin algorithms were associated with a reduction in antibiotic duration and in the ED it was associated with a reduction in the proportion of adults receiving antibiotics.<br><br>Using Procalcitonin algorithms resulted in cost-savings in children within the ED and adult patients within the ICU. Procalcitonin-guided treatment was shown to have a probability of ≥ 84% to be cost-effective for all hospital settings and populations.	ICU<br>ED
Yeung et al. (2015)	The implementation of a regional Clostridium difficile infection (CDI) management policy with Clinical Pharmacy and Medical Microbiology and Infection Control involvement would lead to an improvement in concordance in prescribing practices to a CDI disease severity assessment and pharmacological treatment algorithm in patients diagnosed with CDI at a major Canadian teaching hospital	Two-phase quality assurance study	391 Clostridium difficile infection episodes	Due to timelines, we did not assess the quality of the evidence for single studies.	N/A	Adherence to the Clostridium difficille infection treatment algorithm was higher in post-implementation of the algorithm compared to pre-implementation.<br><br>The median time to treatment with vancomycin was reduced from five days to one day, with median length of hospital stay decreasing from 30 days to 21 days post-implementation.<br><br>There was no difference in 30-day all-cause mortality.<br>Implementation of a regional CDI management policy<br>Number of treatment concordance with correct route of antibiotics: pre-implementation of management policy, 112 (79.4%); post-implementation of management policy, 245 (86.6%).<br>Number of treatments with correct duration: pre-implementation of manangement policy, 58 (41.1%); post-implementation of the manangement policy: 146 (51.6%).	Patient<br>Tertiary care teaching hospital

First Author (year)	Outcomes	Type of Study	Number of participants/or number of studies included	Certainty of the evidence (high, moderate, low)	Measure of effect	Main findings	Comments (study population)
Backman, C. (2011)	Analysis of infection control programs	Systematic review of 32 studies including:<br>27 interrupted time series<br>3 pre- and post-intervention without a control group<br>2 pre- and post-intervention with a control group.	N/A	The quality was not assessed using standardized quality appriasal tools	N/A	The interventions in the included studies were assessed using the tier 1/tier 2 framework.<br>18 (56.25%) studies had an administrative measure as an intervention;<br>20 (62.5%) studies had education and training of health care personnel;<br>8 (25.0%) studies had judicious use of antimicrobial agents;<br>17 (53.1%) studies used surveillance;<br>24 (75.0%) studies had infection control precautions to prevent transmission;<br>7 studies (21.9%) introduced environmental measures;<br>9 (28.1%) studies used patient decolonization.	Practitioner<br>Patient
Blinova, E. (2013)	The rate of documented infections<br>Prevalence of antimicrobial use among pediatric patients admitted to the PICU.	Prospective point-prevalence study	113 participants	Due to timelines, we did not assess the quality of the evidence for single studies.	N/A	Forty-two of 60 patients (70%) received antimicrobials in period A and 42 of 53 patients (79%) received antimicrobials in period B.<br>Of the patients on antimicrobials, 45% in period A and 52% in period B had a diagnosis of infection. Pneumonia and sepsis were the most common infections in period A, whereas pneumonia and other respiratory tract infections were the most common in period B.<br>Antimicrobials were commonly prescribed for documented infection (38%) during period A and empiric therapy (47%) during period B. Cefazolin, cefuroxime, vancomycin, and gentamicin were the commonly used antimicrobials during both periods.<br>Inappropriate antimicrobial use ranged from 16.7% to 61.9%, depending on assessors and surveillance period. The most common reasons for inappropriate use were overly broad spectrum, wrong dosage, and unwarranted overlap of spectrum.	Patient<br>Tertiary care pediatric teaching hospital in Toronto, Canada
Elligsen, M. (2012)	Use of targeted antimicrobials in the level III intensive care units (ICUs)<br>Use of targeted antibiotics within each of the 3 individual ICUs<br>Nontargeted antibiotic use<br>Overall antibiotic use (the sum of targeted and nontargeted agents) measured in days of therapy per 1,000 patient-days across the units as well as overall drug acquisition costs	Prospective controlled interrupted time series analysis	4,697 participants:<br>pre-intervention: 2,358 participants<br>Post-intervention: 2,339 participants	Due to timelines, we did not assess the quality of the evidence for single studies.	N/A	About 34% of physician antibiotic prescription orders were suggested by a stewardship program team to be optimized. The most common suggestion was to discontinue the antibiotic and the most common reason for physicians to reject the suggestion was the suspicion of other infections or additional pathogens.<br><br>The stewardship program resulted in a decreased mean monthy broad-spectrum antibitoic use as well as a decrease in the duration of antibiotic therapy and the use of targeted antimicrobals immediately after the intervention was implemented.<br><br>Within the time series model, the use of these same targeted antimicrobials did not change in the medical and surgical units that did not receive the audit and feedback intervention.<br><br>Overall antibiotic use decreased from 1,134 days of therapy per 1,000 patient-days in the preintervention period to 985 days of therapy per 1,000 patient-days in the postintervention period ($P = .003$). This was driven by decrease of targeted antibitics, use of non targeted did not change. Antibiotic expenditures decreased by $95,000 in the postintervention period.<br><br>Significant increase in overall gram-negative susceptibility to meropenem in the postintervention period, compared with the preintervention period. Susceptibility of ceftriaxone, piperacilin-tazobactam, ciprofloxacin and ceftazidime remained unchanged.	Patients
Flokas, M. E. et al (2017)	Prevalence of antibiotic treatment.	Systematic review.	30 studies (14 prospective and 16 retrospective cohort).	Moderate-assessed by the study: majority of studies were given a rating 6, and lowest was a rating of 5. Quality was judged based on Newcastle–Ottawa Quality Assessment Tool with a maximum score of 6.	Odds ratio.	Being of female sex increased the odds of receiving treatment for asymptomatic bacteriuria. Additonally, overinterpretation of laboratory results were associated with increased odds of receiving antibiotic treatment for ASP. Female sex (OR = 2.11, 95% CI, 1.46–3.06)<br>Laboratory results: 1.<br>Isolation of gram-negative pathogens (OR=3.58, 95% CI, 2.12–6.06)<br>2. Pyuria (OR = 2.83, 95% CI, 1.9–4.22) 3.<br>Nitrite positivity (OR=3.83, 95% CI, 2.24–6.54).	Provider<br>Patient
Fontela (2017)	Describe the criteria that currently guide empiric antibiotic treatment in children admitted to Canadian PICUs.	Cross-sectional survey.	99 physicians.	High.	N/A.	With the exception of a positive viral polymerase chain reaction, our findings suggest that physicians rarely consider reducing the duration of antibiotics despite clinical improvement. In contrast, they will prolong the duration when faced with a nonreassuring characteristic.	Provider
Goldman, R. D. et al. (2009)	Extent of practice variations among pediatric emergency department practitioners	Prospective<br>Concurrent cohort study of consecutive infants	257 participants	Due to timelines, we did not assess the quality of the evidence for single studies.	N/A	There were significant differences between each tertiary hospital including differences in drug administration, as well as varitions between pediatric ED physicians. There was also no endorsed guideline that provided recommendations on prescriptions for infants.<br><br>Nonadherence to available guidelines due to:<br>1. Experience and confidence in diagnosing serious bacterial infections in young infants<br>2. Providers and practice locations accounted for small proportions of practice variability, whereas differences in clinical presentation and illness severity underlay much of observed practice variability<br><br>Even though they were trained PEM physicians, the study still showed significant practice variations contradicting the idea that it is due to lack of homogenous training.	Provider<br>Tertiary pediatric EDs
Krockow et al. (2019)	Threat perceptions associated with antimicrobial resistance<br>Perceived benefits and barriers associated with antibiotic stewardship<br>Physicians perceived broad-spectrum antibiotics to be effective and low risk.	Qualitative systematic review	34 studies	The quality was not assessed using standardized quality appriasal tools	N/A	The risk of antimicrobial resistance was generally perceived to be serious, while prescribers believed in the benefits of optimizing prescribing, the direct link between over-prescribing and antimicrobial resistance was questioned, and prescribers' behaviour change was frequently considered futile when fighting the complex problem of antimicrobial resistance.	Provider
Kuster, S. P. et al (2014)	Antimicrobial susceptibility<br>Risk of resistance after exposure	Prospective surveillance study	4062 episodes	Due to timelines, we did not assess the quality of the evidence for single studies.	Odds ratio	Clinical information and antimicrobial susceptibility were available for 4062 (90%) episodes; 1193 (29%) of episodes were associated with receipt of 1782 antibiotic courses in the prior 3 months. The study found that selection for antibiotic resistance was class specific. Further, time elapsed since most recent antibiotic was inversely associated with resistance. Risk of resistance after exposure declined most rapidly for fluoroquinolones and penicillins and reached baseline in 2–3 months. The decline in resistance was slowest for macrolides, and in particular for azithromycin. There was no significant association between duration of therapy and resistance for any antibiotic class.	25 hospitals<br>19 laboratories<br>85 long-term-care facilities
McKay, R. et al (2016)	Antibiotic prescription	Systematic review	28 studies:<br>Retrospective<br>Prospective<br>cross-sectional	**Good or high quality**<br>The quality was assessed using a tool developed by the authors based on the SIGN 50 (Scottish Intercollegiate Guidelines Network) for cohort and case-control studies, as recommended by a review of quality assessment tools (18), as well as incorporating elements of the Quality Assessment Tool for Observational Cohort and Cross-Sectional Studies from the National Institutes of Health's National Heart, Lung, and Blood Institute	Odds ratio	On a provider level, factors that were associated with higher odds of antibiotic prescribing included features on physical exam (fever, purulent sputum, abnormal respiratory exam, and tonsillar exudate), and physician perception of patient's desire for antibiotics (range of aORs:2.11–23.3). Patient's desire for an antibiotic prescription was not associated or was modestly associated with physician prescribing(range of adjusted ORs: 0.61–9.87) . A diagnosis of acute bronchitis was also associated with increased antibiotic prescribing(range of adjusted OR: 1.56 – 15.9).<br>On a patient level, one study showed that the male sex had a higher odds of receiving an antibiotic prescription which was statistically significant. 10 studies implied that older people had higher odds of receiving an antibiotic prescription compared to younge people, whereas three studies showed the opposite.	Provider<br>Patient
Tan, C. et al (2017)	The variability, and risk-adjusted variability, in antibiotic use across acute care hospitals in Ontario	Registry-based study<br>Province-wide analysis	129 unique institutions:<br>17 teaching hospitals<br>50 large community hospitals<br>39 medium community<br>23 small community hospitals	Due to timelines, we did not assess the quality of the evidence for single studies.	N/A	There was 7.4-fold variability in the quantity of antibiotic use across the 129 acute care hospitals, from 253 to 1873 DDDs/1000 PDs. Variation was evident within hospital subtypes, exceeded that explained by hospital and patient characteristics.<br><br>Small community hospital-type was the strongest predictor of overall antibiotic use, associated with a 2.4- fold increase in consumption compared with large community hospitals (95%CI 1.39–4.13). Teaching hospitals were also associated with greater utilization (relative risk 1.17; 95% CI 1.01–1.36).<br><br>The proportion of short- and long-stay admissions was associated with reduced antibiotic use, with each 10% increase in admissions < 2 or>10 days associated with decreased antibiotic use by 30%and 50%, respectively.	N/A
Warreman et al. (2019)	Antibiotic prescription behavior	Systematic review	4046 participants	The quality was assessed using the Critical Appraisal Skills Program (CASP) 2017 scoring system for quality assessment of qualitative research. There was no overall assessment provided.	N/A	Determinants that influenced the physician's antibiotic prescription behavior included the examples set by colleagues, social team dynamics, hierarchical influence and reputational risk.<br>Logistics (time to decision/ time pressure), benevolence, applicability of guidelines, complaints and litigation, professional autonomy, prior experience, clinical experience and education, tolerance of uncertainty, perceived ability to communicate the decision, and fear of adverse outcomes also influenced antibiotic prescription behavior.	N/A

Grey Literature (country or organization and access date)	Website	Access date	Document Characteristics (budget, policy area, guideline/rules, goals, policy evaluation, research)	Setting/target audience	Description/Aim	Key findings, recommendations or messages	Studies that evaluated the program/intervention
Choosing Wisely Canada	https://choosingwiselycanada.org/campaign/antibiotics/<br>https://choosingwiselycanada.org/?s=Antimicrobial+resistance	Accessed: March 23, 2019	Guidelines/rules	Physicians and general public	The main goal of the campaign, *Using Antibiotics Wisely* , is to reduce unnecessary use, and promote appropriate use of antibiotics in Canada, through education and awareness.	The campaign provides informational posters, evidence-based tools, and patient materials that promote and educate about appropriate use of antibiotics. In primary care, about 30-50% of antibiotics prescribed for acute respiratory infections are unnecessary.	Silverstein W, Lass E, Born K, Morinville A, Levinson W, Tannenbaum C. A survey of primary care patients' readiness to engage in the de-adoption practices recommended by Choosing Wisely Canada. BMC research notes. 2016;9:301.
Public Health Ontario	https://www.publichealthontario.ca<br>https://opha.on.ca/Home.aspx<br>https://www.publichealthontario.ca/en/health-topics/antimicrobial-stewardship	Accessed: March 23, 2019	Guidelines/rules	Physicians	The *'Antimicrobial Stewardship Strategy: Empiric Antibiotic Prescribing Guidelines'* provides overview of recommendations to improve selection of appropriate treatment for common infectious diseases.	The guidelines recommend that physicians should choose a therapy that is based on the site of infection, the common pathogens encountered, the local epidemiology and resistance patterns, evidence and clinican consensus, antimicrobial stewardship principles, formulary availability, and antimicrobial costs.	N/A
Saskatchewan	https://www.saskatchewan.ca<br>http://www.rqhealth.ca/departments/antimicrobial-stewardship-program	Accessed: March 23, 2019	Guidelines/rules	Healthcare professionals, administration and policy makers	The Antimicrobial Stewardship Program is an interdisciplinary collaboration that promotes optimal use of antimicrobials, including determination of the need for antimicrobials, and if needed, selection of the right drug, dose, duration and route of administration. It is an evidenced-based practice with the primary goal of reducing rates of antimicrobial resistance.	The campaign provides pamphelts and videos promoting better antibiotic use and guidance.	N/A
Manitoba	https://www.gov.mb.ca/index.html<br>https://www.gov.mb.ca/health/publichealth/	Accessed: March 23, 2019	Guidelines/rules	Regional Health Authorities (RHAs) and other health care facilities/organizations.	Prevention and Control of Antimicrobial-Resistant Organisms	The guiding principles used in developing these guidelines include:<br>1. Limiting transmission of antimicrobial-resistant organisms within all health care settings.<br>2. Minimizing development of infections with antimicrobial-resistant organisms in patients in all health care settings.<br>3. Promoting patient safety in all health care settings.<br>4. Achieving these goals in a fiscally responsible manner.<br>5. Providing evidence – based best practice recommendations.<br>6. Updating the antimicrobial-resistant organism guideline in a timely manner, as required.	N/A
WHO	https://apps.who.int/iris/bitstream/handle/10665/193736/9789241509763_eng.pdf?sequence=1	Accessed: March 4, 2019	Guidelines/rules	National governments	Objective 1) Improve awareness and understanding of antimicrobial resistance through effective communication, education and training.<br>Objective 2) Strengthen the knowledge and evidence base through surveillance and research<br>Objective 3) Reduce the incidence of infection through effective sanitation, hygiene and infection prevention measures.<br>Objective 4) Optimize the use of antimicrobial medicines in human and animal health<br>Objective 5) Develop the economic case for sustainable investment that takes account of the needs of all countries, and increase investment in new medicines, diagnostic tools, vaccines and other interventions	This agreed framework will be used by the member states to create their own national plans to achieve the goals outlined by the global action on antimicrobial resistance.	N/A
ECDC	https://ecdc.europa.eu/en/publications-data/directory-online-resources-prevention-and-control-antimicrobial-resistance-amr	Accessed: March 4, 2019	Guidelines/rules	Healthcare professionals, administration and policy makers	Provides a central directory for the prevention and control of AMR with courses, relevant policy briefs, and guidance.	The ECDC takes a holistic approach by providing guidance on how to limit infections by microorganisms and healthcare proceudres, reducing antibiotic use, requirements to be adhered to by healthcare settings, strategies and Europe wide action plans.	N/A
International Federation of Pharma Manufacturers & Associations<br>(IFFMA)	https://www.ifpma.org/subtopics/antimicrobial-resistance/	Accessed: March 4, 2019	Guidelines/rules	Healthcare professionals, administration, policy makers, and pharmaceutical companies	IFFMA work to bring industry and broader health community together to foster innovation, promote resilient regulatory systems and high standards of quality, uphold ethical practices, and advocate sustainable health policies to help address the global antibiotics crisis.	IFFMA had published guidelines and inforgraphics outlining strategies to educate patients and healthcare providers, alternative ways of tackling infections, and preventing infections. The IFFMA has also published a timeline for pharmaceutical companies to combat antimicrobial resistance.	N/A
CDC Centre for Disease Control and Prevention	https://www.cdc.gov/drugresistance/solutions-initiative/index.html	Accessed: March 4, 2019	Guidelines/rules	Healthcare professionals, administration, agriculture industry and policy makers	The CDC seeks to tackle the threat of antibiotic resistance by mobilizing action and helping promote aggressive action with healthcare, veterinary, and agriculture partners nationwide. The CDC also seeks to empower the United States of America to respond comprehensively, efficiently, and effectively to this crisis.	The CDC has come up with a multi-prongued approach where they hope to: 1) detect, respond, and contain resistant pathogens with the state of the art lab techniques and treatments. 2) prevent the spread of resistant infections with careful monitoring and reducing antibiotic use. 3) Encouraging innovation not only in detection and reduction in antibiotic use, but also in prevention of such infections and developing new treatments and diagnostic tests.	N/A
United Kingdom	https://www.gov.uk/government/news/30-million-of-funding-to-tackle-antimicrobial-resistance<br>https://www.rcgp.org.uk/clinical-and-research/resources/toolkits/target-antibiotic-toolkit.aspx<br>https://www.ukri.org/research/themes-and-programmes/tackling-antimicrobial-resistance/	Accessed: March 8, 2019	Guidelines/rules	Healthcare professionals	The government of the united kingdom hopes to accomplish several goals, these include: 1) Using antimicrobials more sparcely and in a more targeted and efficient manner. 2) Investing in research and education on AMR.	The government has developed a toolkit, called Target, to help guide clinicians and patients with the information that they need. TARGET stands for: Treat Antibiotics Responsibly, Guidance, Education, Tools. It includes a range of resources that can each be used to support prescribers' and patients' responsible antibiotic use, helping to fulfil CPD and revalidation requirements. This is reviewed every 2-4 years. The government also maintains records and makes consumption and prescription information publicly available. This was last done in 2013.	N/A
